# Microangiopathic Hemolytic Anemia in 57-year-old Woman with Borderline Serous Tumor of the Ovary: Real-Time Management of Common Pathways of Hemostatic Failure

**DOI:** 10.4084/MJHID.2012.030

**Published:** 2012-05-06

**Authors:** Gloria J Morris, Henry C Yaeger, Francis Hamm, Sibyl Irwin, Salvatore J Scialla

**Affiliations:** 1 From the Mount Sinai Hospital of Queens, Long Island City, NY;; 2 the Departments of Nephrology; 3 Obstetrics and Gynecology, and; 4 Pathology, Moses Taylor Hospital, Scranton, PA; and; 5 Hematology & Oncology Associates of NE PA, Dunmore, PA

## Case Study

**Figure 1.**
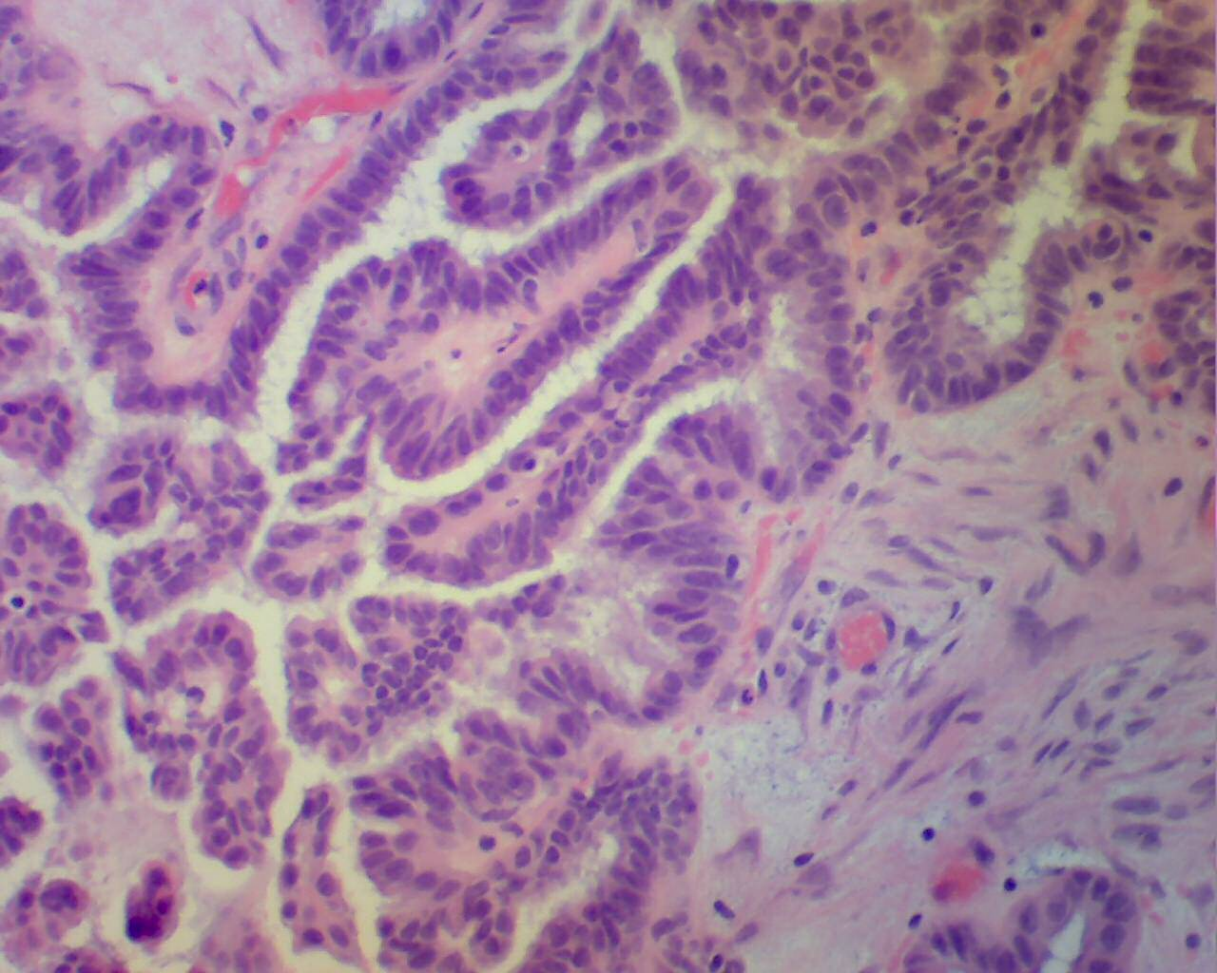
H+E stained sections of right ovary showing borderline serous tumor. Courtesy of Dr. Sybil Irwin, Department of Pathology, Moses Taylor Hospital, Scranton, PA.

**Figure 2.**
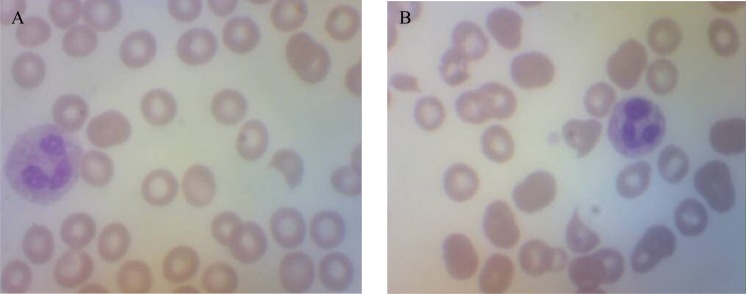
H+E stained peripheral blood smear showing shistocytes, 100X power. (a) POD (post-operative day) #3, (b) POD#4. Courtesy of Dr. Sybil Irwin, Department of Pathology, Moses Taylor Hospital, Scranton, PA.

A 57-year-old female with history of myocardial infarction and need for cardiac catheterization with stent placement requiring clopidogrel for many years, as well as a history of hypertension requiring lisinopril, was evaluated by her gynecologist for ongoing pelvic pain. She had no personal history of rheumatologic disorders, including systemic sclerosis. She had no family history of cancers. She underwent a pelvic ultrasound which showed bilateral complex ovarian cysts. She was slated for a laparoscopic bilateral salpingo-oophorectomy (BSO), and clopidogrel was stopped 2 weeks prior to surgery. Pre-operative CA-125 level was normal, as was her complete blood count, hepatic, and renal function. Upon laparoscopy, there was found to be enlargement of the right ovary with excrescences, grossly suspicious for malignancy, thus requiring exploratory laparotomy. Frozen section of the right ovary described a borderline tumor, and a hysterectomy was then performed in addition to the BSO. Estimated blood loss was 350 cc. The final pathology confirmed a serous Borderline tumor without microinvasion or evidence of invasive carcinoma in either ovary (**Figure 1**). Postoperatively the patient was given thromboembolism prophylaxis with heparin 5000 U subcutaneously every 8 hours. She developed fevers to 101 degrees F for 2 days without an obvious source of infection; blood and urine cultures were negative, and chest x-ray was unremarkable, with the exception of atelectasis. Empiric antibiotics however, were added. Neurologic examination was unremarkable and the patient was lucid without evidence of mental status changes. Creatinine levels rose to 2.0 mg/dL, thought due to hypovolemia, and intravenous fluids were increased. A CBC showed decrease in the platelet count to 110 K/ul (ref 142–424) on post-operative day (POD)#2, and to 63 K/ul on POD #3, when the patient was evaluated by a hematologist. The hemoglobin (Hgb) level was 8.1 g/dL (ref 12.2–16.2), prothrombin time (PT) 13.1 seconds (ref 11.7–14.7 sec), fibrinogen 550 mg/dL (ref 188–421 mg/dL), lactate dehydrogenase (LDH) 2764 U/L (ref 313–618 U/L), haptoglobin level <7 mg/dL (ref 42–312 mg/dL), and evaluation of the red cells by peripheral blood smear with 1–2 shistocytes per high-powered field (HPF) (**Figure 2a**). D-dimer level (fibrin-degradation split products) was 2.68 ug/ml (ref 0.0–0.42 ug/ml). Cr 1.0 mg/dL (ref <1.2 mg/dL), which was improved from 2.0 mg/dL with the addition of further IV fluids on POD#4. The patient then developed worsening blood pressure, requiring antihypertensives including clonidine, and experienced chest pain with swelling in her legs; workup including CT angiogram and an ultrasound of the lower extremities with Doppler negative for thromboembolism. Heparin was stopped and an interim diagnosis of heparin-induced thrombocytopenia (HIT) was considered; a prophylactic argatroban infusion was given while HIT studies were pending and was continued empirically for 5 days. On POD#4, platelets were 67 K/uL, Hgb 8.1 g/dL, LDH 2964 U/L, with 3–5 shistocytes per HPF (**Figure 2b**), with D-dimer (FDP) level 2.25 ug/ml She was neurologically intact and lucid. The patient was placed on prednisone and platelets improved to 83 K/uL on POD#5 with hemoglobin 7.7 g/dL requiring red cell transfusion, LDH 1694 U/L, fibrinogen 556 mg/dL, with liver function testing normal. HIT ELISA was negative as was heparin-induced platelet aggregation assay, and there was no evidence LAC. Factor VIII levels were followed, showing normal levels with no evidence of inhibitor, ristocetin cofactor activity was normal, and ADAMTS13 level. Creatinine level was then stable. The patient was observed on prednisone and treated empirically for fevers. On POD #6, the platelet count rose to 106 K/uL, hemoglobin to 7.9 g/dL, requiring additional red cell transfusion, LDH stable at 1610 U/L, with examination of the peripheral blood smear showing persistently 4–5 shistocytes per HPF, and creatinine level of 1.2 mg/dL. On POD#7, hgb level 9.7 g/dL, platelets 177 K/ul, LDH 1618 U/L, Cr 1.2 mg/dL, fibrinogen 301 mg/dL. On POD#8, the platelet count rose to 222 K/ul, hemoglobin to 10.2 g/dL, LDH 979 U/L, peripheral smear with 3–4 shistocytes per HPF, and stabilized for the rest of the hospitalization. The differential diagnosis included microangiopathic hemolytic anemia (MAHA) associated with thrombotic thrombocytopenic purpura (TTP)/hemolytic-uremic syndrome (HUS), systemic inflammatory response syndrome (SIRS), antiphospholipid antibody syndrome, a compensated disseminated intravascular coagulation (DIC), and /or hypertension-associated MAHA. In looking for specific trigger, we also asked the question as to whether MAHA has been triggered by ovarian tumors.

## Discussion

Microangiopathic hemolytic anemia (MAHA) is historically described as a group of clinical disorders characterized by fragmentation of the red blood cells in the circulation and resultant intravascular hemolysis, occurring through fibrin deposits inside the lumens of arterioles and capillaries or through damaged epithelium and vessel walls.^1^ Widespread microthrombi occur in arterioles and capillaries and can catalyze thrombocytopenia, especially during two prime disorders, thrombotic thrombocytopenic purpura (TTP) and hemolytic-uremic syndrome (HUS), which results in severe MAHA, for which a major clinical suspicion must always be ruled out. MAHA may be observed in patients who experience sepsis, disseminated carcinomatosis, disseminated intravascular coagulation, catastrophic antiphospholipid antibody syndrome (APS), organ transplantation, complications of pregnancy, malignant hypertension, and exposure to venoms, toxins, or antineoplastic agents such a mitomycin-C or cyclosporine.^2^–^8^ Clopidogrel has previously been reported to cause TTP but usually this has been reported to occur within the first 2 weeks of initiation of the treatment.^9^,^10^ Typically the classical pentad of symptoms associated with TTP are fevers, central nervous systems changes, hemolytic anemia with shistocytes on a peripheral blood smear and associated with elevated LDH, renal insufficiency, and thrombocytopenia. Clinically, only a triad of the latter three are sufficient for a clinical diagnosis, which is curable with aggressive plasma exchange and pheresis.^11^–^14^ Deficiency of ADAMTS13 von Willebrand factor-cleaving metalloprotease has been implicated in the pathogenesis of acute recurrent TTP, probably resulting from the combination of this deficiency due to autosomal recessive trait with decrease synthesis, intersecting with the mechanism of endothelial cell damage.^15^ However, the clinical picture common to TTP and HUS may overlap with DIC or SIRS in a patient presenting with fevers and thrombocytopenia.^16^ In addition, catastrophic antiphospholipid antibody syndrome, which is an immune reaction and “symptom-complex” difficult to distinguish from TTP-HUS, can also present with renal failure, encephalopathy, and even acute respiratory distress syndrome, and can catalyze a hypercoagulable state.^17^ Hence, when faced with these clinical signs, how does the consultant sort them out?

We report another confounding trigger to the hemolytic cascade presented here. Tumor-induced MAHA is also associated with invasive and advanced malignancy,^18^ and only one case report has linked this complication with a benign pelvic tumor.^19^ Overexpression of urokinase-type plasminogen activator in ovarian cancer cells may lead to hypercoagulability.^18^ Of paraneoplastic syndromes associated with gynecologic malignancies, DIC is of the most common,^2^,^3^,^20^–^23^ probably caused by shearing of RBCs on fibrin strands from contact with tissue factor or with tumor cell emboli, a mechanism similar to that seen in mucin-releasing GI malignancies.^24^,^25^ Acute promyelocytic leukemia also causes DIC and MAHA through release of procoagulant,^26^ a mechanism also reported in rhabdomyosarcoma as a specific and trackable factor VIII antigen homolog which can remit and relapse with disease progression,^27^ as can increased platelet surface sialytransferase activity in other cancers.^28^ Even mechanical heart valves can cause MAHA with raising of FDP levels after shearing of RBCs.^29^,^30^

How the clinician should proceed analytically is pivotal in deciding upon which intervention should be made in a timely and life-saving fashion. In the assessment of anemia and thrombocytopenia in the postoperative or intensive care setting careful history is required related to prior hematologic disorders, onset of disorder, location of bleeding, concurrent illness, and drug exposure. Pre-existing organ failure may help the clinician to sort out these processes from acute MAHA with new onset organ failure.^17^ Physical examination is crucial to determining likelihood of acute infection and/or neurologic changes, as well as evidence of new organ failure such as anuria or oliguria, to evaluate for sites of bleeding and/or bruising due to hemolysis or thrombocytopenia, and to evaluate for physical signs suspicious for thrombosis. Evaluation of the coagulation profile including PT, PTT, fibrinogen, D-dimer (fibrin-split products), and lactate dehydrogenase (LDH), including fractionation of isoenzymes, is crucial in order to formulate a differential diagnosis.^17^ Measurement of Factor VIII levels may be helpful in determining extent of consumption in DIC versus compensatory mechanisms activated thus elevating the levels due to systemic inflammation; however, immediate quantitation of this level may not be readily available in all hospital laboratory settings. Visual assessment of the peripheral blood smear for the morphology of red blood cells to detect schistocytes or red cell fragments, presence of platelet clumping which may be related to chelating without EDTA versus lack of platelets produced. Once acute thrombocytopenia is confirmed on the blood smear, HIT as a severe immune complex-mediated syndrome caused by interaction between heparin and heparin-induced platelet factor-4 should be considered, especially if the hallmarks of HIT are seen including a sudden decrease in the platelet count by 50% of decrease from normal to less than 100,000 per ul is seen in the presence of heparin;^31^ heparin therapy should be stopped immediately with an alternative to anticoagulation initiated such as direct thrombin inhibitors argatroban or lepirudin. Red blood cell transfusions may be indicated if blood counts drop severely below a level which causes clinical instability; but platelet transfusions are not recommended in literature guidelines until a firm diagnosis is established, as they are known to aggravate thrombosis in HIT as well as TTP-HUS by adding to already activated platelet-thrombin complexes, and are of no proven benefit in immune thrombocytopenic purpura unless there is evidence of acute bleeding.^32^

## Conclusion

We present here a case of a 57-year-old woman previously on clopidogrel, who experienced MAHA after surgery for an ovarian mass which was deemed pathologically as a borderline serous tumor. Her baseline CBC, liver and renal functions were normal. However, post-operatively she experience fevers, renal insufficiency, hypertension, and MAHA with shistocytes and elevated LDH as well as thrombocytopenia, all suspicious for TTP, but further sorting of clinical information led to other differential diagnoses, including fever from atelectasis or SIRS, with elevated fibrinogen as in acute phase reaction; renal insufficiency from hypovolemia, as this responded to fluids; the possibility of malignant hypertension; and the possibility of DIC triggered from the release of intracellular contents from a necrotic ovarian tumor.

Multiple triggers of the coagulation system can lead to a common pathway of hemostatic failure that incorporates the systemic inflammatory response, leading to multiple organ failure. The specific criteria seen in DIC, TTP, HIT, and catastrophic APS seem to overlap. The key maneuver is determining the trigger (i.e., infection or medication), and tracking possible and available hemostatic markers through the clinical course, such as plasma thrombomodulin (TM), antithrombin, protein C levels, which are elevated (the former) or markedly reduced (the latter), especially in patients with DIC and TTP.^33^ On initial presentation there is an often an overlapping of differential diagnoses and possible interventions. Some common characteristics may include the activation of the coagulation system from direct contact of tumor cells with tissue factor, vis a vis, with elevated and qualitative Factor VIII, MAHA from fibrin stranding, or the presence of antiphospholipid antibodies. The difficult task is embarking on the correct and logical hematologic intervention(s), whether anticoagulation, steroids, transfusion, or aggressive plasma exchange; while life-saving in HUS/TTP, the latter may pose cardiovascular risk in some patients significant enough to consider the short-term risk versus benefit ratio. This case demonstrated a scenario of multiple possible triggers for MAHA, all of which may overlap clinically. Here, markers including elevated factor VIII levels suggested inflammatory response over other consumptive processes; however, as these entities were further being investigated for their confirmation, the patient was initially treated with a combination of alternative anticoagulation, a trial of steroids, empiric antibiotics, and observed for other signs of multiorgan failure. Clinical judgment must then prevail in staving off a potentially tenuous situation, guarding toward possible clinical compromise requiring aggressive intervention, versus one that may be treated supportively based upon the underlying disorder.

**Figure 3.**
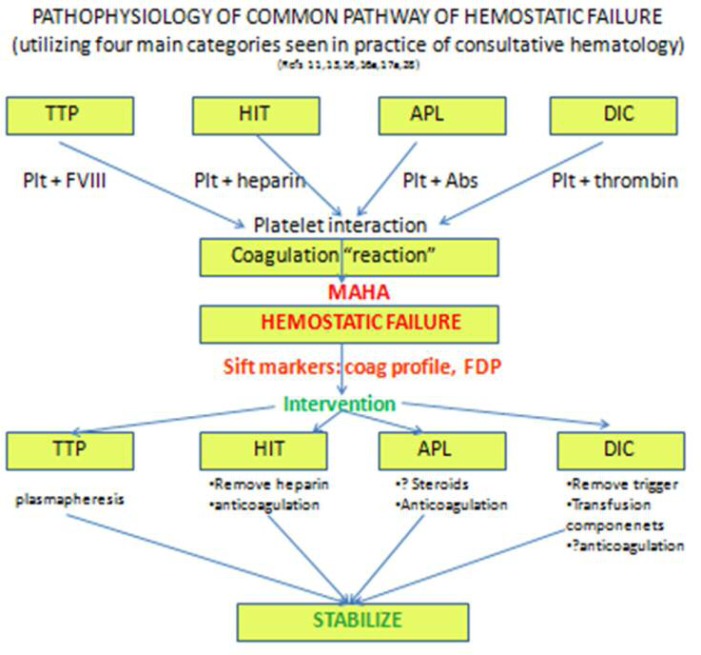
Flow diagram of proposed analytical decision-making in cases of hemostatic failure, which stem from multiple possibilities in a differential diagnosis; while these converge at a common pathway marked by overt hemostatic failure, pursuit of a final diagnosis guides specific intervention. Abbreviations: Abs=antibodies; APL= antiphospholipid antibody syndrome; DIC=disseminated intravascular coagulation; FDP=fibrin degradation products; MAHA=microangiopathic hemolytic anemia; HIT = heparin-induced thrombocytopenia; plt=platelet; TTP= thrombotic thrombocytopenic purpura.

In **Figure 3**, we summarize and propose a chronologic and strategic approach for the clinician who is faced with this dilemma of post-operative MAHA with thrombocytopenia and renal insufficiency with a large differential diagnosis including TTP, HIT, APL, and DIC. (a) Careful history must be taken with attention the onset of change in the CBC, induction of hemolysis, and consumption of platelets, concomitant with drug exposure, and put into clinical context; abrupt onset may suggest acute change, and a list of drugs may point toward more specific inciting mechanisms. (b) Physical examination with attention to hematologic manifestations such as purpura, thrombosis, and hemorrhage may help to distinguish the severity of the condition and would guide toward the absolute need for anticoagulation. (c) Immediate laboratory investigation of coagulation profile to distinguish DIC from other hemostatic disorders, including APL with the elevation of PTT alone and presence of associated antibodies, or a more normal coagulation profile in which platelet consumption may preside. (d) Visual assessment of blood cell morphology for red cell fragmentation or platelet aggregation is essential. Further distillation of the pathogenesis of hemostatic failure is illustrated as the final ability of platelets to interact with larger blood vessels, and to form a clot with sustainable integrity. The above described “markers” of impending hemostatic failure can help to determine which entity in the differential diagnosis to more fully pursue, and which specific intervention to follow, including plasmapheresis for TTP; anticoagulation for APL or even DIC (or its alternative for HIT); or treatment of the underlying cause with support from indicated transfusions for DIC. In this particular situation, a SIRS-like phenomenon was felt to prevail, and did not require a more intense intervention, but a successful supportive approach.
